# Forensic dentistry - Present and future

**DOI:** 10.6026/973206300222680

**Published:** 2026-04-30

**Authors:** Sonali Khatri, Sneha Masne Deshpande, Pawan Rebello, Nidhi Sinha, Pallavi Khare, B Vidya, Deeksha Goel

**Affiliations:** 1 Department of Oral Pathology and Microbiology, Genesis Institute of Dental Sciences and Research, Firozpur, Punjab, India; 2 Department of Oral Pathology and Microbiology, Bharati Vidyapeeth (Deemed to be University) Dental College and Hospital, Belapur, Navi Mumbai, Maharashtra, India; 3 Department of Oral & Maxillofacial Pathology & Oral Microbiology, Sardar Patel Post Graduate Institute of Dental and Medical Sciences, Lucknow, Uttar Pradesh, India; 4 Department of dentistry, Dr. Harvansh Singh Judge Institute of Dental Sciences & Hospital, Panjab University, Chandigarh, India; 5 Department of Conservative dentistry and Endodontics, DJ College of Dental Sciences and Research, Modi Nagar, India

**Keywords:** Forensic dentistry, artificial intelligence (AI), machine learning (ML)

## Abstract

Evolved from the Latin word "Forensis", meaning forum or the court of law, forensic science branches out into a vital subfield, forensic dentistry, which is dedicated to the application of science for the preservation and presentation of dental evidence in the interest of justice. Forensic dentistry is transitioning from manual morphology to high-precision digital diagnostics. While rooted in the thermal resilience of dental tissues, the field now leverages convolutional neural networks (CNNs) for automated age estimation and DNA methylation for epigenetic profiling. Therefore, it is of interest to evaluate the shift toward the Internet of Dental Things (IoDT) and 3D digital twinning via CBCT for immutable identification. By synthesizing 2024-2026 technological advancements, we provide a roadmap for enhancing the objectivity and legal admissibility of dental evidence. These molecular and AI-driven tools are critical for modernizing human identification in mass disaster and criminal investigations, offering superior speed and accuracy over traditional comparative methods. Thus, we report the pivotal shift from manual dental morphology to AI-driven digital diagnostics.

## Background:

Forensic odontology is an upcoming branch in dentistry where the knowledge of dental and orofacial structures is applied to legal inquiries [1, 2]. It involves the appropriate processing, inspection and assessment of dental evidence, followed by the proper presentation of conclusions in court [3]. Its key purpose is identification of human remains using teeth when bodies are severely disfigured, mutilated or burned beyond recognition. It is necessary in situations where visual identification is impossible, such as in mass disasters like tsunamis and airplane crashes [4]. Recent data from 2023 to 2026 marks dental identification as the most reliable practice in multi-victim disaster scenarios globally. This reliability is due to the high thermal resistance of enamel along with the inherent uniqueness of individual dental patterns [5, 6 and 7]. The pillars supporting identification based on reconstruction are the evaluation of the "Big Four": age, sex, race and stature [4, 9]. The discipline's f uture integrates digital imaging, artificial intelligence and molecular techniques such as epigenetics and microbiome analysis [10, 11-12]. Therefore, it is of interest to describe the current state of the field, emphasizing the transition from traditional reconstructive methods to advanced digital and molecular technologies.

## History:

The origin of forensic dentistry from isolated incidents dates back to ancient times, with recorded cases as early as 66 AD. In 1775, Paul Revere became known as the first American forensic dentist by identifying a fallen soldier through a silver dental bridge [8]. The first dental evidence in a court of law was presented in the famous Webster-Parkman Case of 1849 by Dr. Nathan Keep. In the 1887 Bazaar de la Charité Tragedy in Paris, the systematic use of dental evidence motivated Dr. Oscar Amoëdo to formalize the field. Known as the "Father of Forensic Odontology", Amoëdo published the seminal work L'Art Dentaire en Medicine Légale in 1898. The identities of Adolf Hitler and Eva Braun were verified using their dental records, radiographs and specific prosthetics in 1945 [7]. Bite mark analysis in the late 20th century and groups like the ABFO further helped set standards and formalize methodologies [9].

## Present practices:

Current forensic odontology centers on three primary areas using both established and continually advancing techniques. Comparative Dental Identification involves Antemortem (AM) and Post-mortem (PM) comparison to confirm human identity [1]. Records are compared using manual and radiographic tools to reach results categorized as positive, possible, insufficient evidence or exclusion [3]. Imaging methods like digital radiography and panoramic X-rays are employed for detailing restorations and anatomical features [6]. Forensic Profiling is utilized when AM records are absent to estimate biological sex and chronological age from remains. Age estimation involves matching developmental charts for children or analyzing regressive root changes and cementum apposition in adults [4, 9]. Sex determination is aided by morphological differences in the skull or through molecular DNA analysis of the dental pulp. Ancillary methods such as cheiloscopy, rugoscopy and ameloglyphics are practiced to support the identification process [2]. Bite Mark Analysis is used in sexual assault and child abuse cases, involving software documentation and salivary DNA collection [5].

## The emerging future trends:

The science is quickly shifting focus to digital and molecular methods to enhance objectivity, speed and legal reliability. AI and machine learning have revolutionized tasks by automating comparative identification and minimizing human observer error. 2024-2026 research shows AI algorithms now estimate dental age with a precision of 0.5 years, outperforming manual parameters [10]. 3D geometric data can now be processed using AI for automated, objective bite mark analysis and pattern matching [5]. Epigenetics analyzes DNA methylation levels in dental pulp to provide a highly accurate "biological clock" for age profiling. Proteomics offers a newer alternative for sex determination through amelogenin peptides in enamel, even in highly compromised samples. Recent studies highlight the oral microbiome and miRNA analysis as stable biometric links to connect individuals to specific evidence [12]. 3D digital twinning via CBCT and "Virtopsy" allows for non-invasive, permanent and physical-duplicate evidence preservation through 3D printing. The Internet of Dental Things (IoDT) integrates smart implants with sensors for real-time, traceable identification data [10]. Automated deep-learning now allows for 3D tooth segmentation and profiling even from low- quality, fragmented CBCT scans and digital twinning provides an immutable forensic record that serves as a permanent biometric for high- risk professionals [11] (Figure 1 - see PDF).

## Conclusion:

Forensic odontology is evolving toward a digital-first paradigm, significantly improving identification reliability in fragmented remains. AI-driven segmentation and proteomic sex determination have bridged major gaps between innovative dental science and courtroom evidence. While high implementation costs and a lack of global AI dataset standards remain barriers, the path toward automation is clear. Future progress depends on validating these molecular tools through extensive multi-ethnic studies to ensure global accuracy. Ultimately, integrating digital twinning with IoDT offers a robust, tamper-proof framework for modern human identification.
